# Physicians’ Opinion on Intraoperative Radiotherapy as a Therapeutic De-Escalation Option in Older Women with Early Breast Cancer

**DOI:** 10.3390/curroncol30030214

**Published:** 2023-02-27

**Authors:** Agnès Tallet, Dominique Rey, Clémence Casanova, Delphine Lecourtois, Marie Bergeaud, Marc-Karim Bendiane, Julien Mancini

**Affiliations:** 1 UNICANCER, Institut Paoli-Calmettes, Department of Radiation Oncology, 13009 Marseille, France; 2 Aix-Marseille Univ, INSERM, IRD, ISSPAM, SESSTIM, Cancer, Biomedicine & Society Group, Ligue 2019 Accredited Team, 13009 Marseille, France; 3 UNICANCER, Unitrad, 75013 Paris, France; 4 APHM, BIOSTIC, Hop Timone, 13005 Marseille, France

**Keywords:** early breast cancer, intraoperative radiotherapy, therapeutic de-escalation, patient-centered care, shared decision-making

## Abstract

Background: Intraoperative radiotherapy (IORT) is a therapeutic de-escalation option in older women with low-risk early breast cancer (EBC). A qualitative study was implemented to describe EBC physicians’ points of view on IORT as a de-escalation option. Methods: Recorded face-to-face and telephone semi-structured interviews were conducted among diverse physicians from seven French comprehensive cancer centers. Interview transcripts were grouped as corpus to construct a typology. Thematic analysis was performed. Results: Positions toward IORT were contrasted between the 16 participating physicians. Five fully supported IORT as a de-escalation option, four were not in favor, and seven had a more reserved or neutral opinion. Points of divergence concerned treatment efficacy, treatment duration, side effects and sequelae, psychological impact, compliance with adjuvant endocrine therapy, logistical constraints, financial cost, and availability of other techniques of partial breast irradiation. Physicians in favor of IORT emphasized direct benefits for the patient, and those against pointed the lack of specific guidelines, risk of lost opportunity in older women with long life expectancy, and challenges of shared decision making. Conclusions: Despite national policies to preserve cancer patients’ quality of life and increase their participation in medical decision making, therapeutic de-escalation using IORT is not consensual among physicians. Further efforts are needed to promote patient-centered care.

## 1. Introduction

Breast cancer is the most common cancer in women, with 48% of cases occurring in women aged over 65 years in France [1]. This cancer is considered to have good prognosis, with an estimated 5-year net survival of 87% [2]. For early breast cancer (EBC), standard treatment is breast-conserving surgery (BCS), followed by whole-breast adjuvant radiotherapy (WBRT) [3]. However, for the purpose of therapeutic de-escalation, different modalities of breast irradiation have been tested over the past 20 years to deliver the optimal dose of radiation to the tumor and reduce toxicity. Different therapeutic modalities are currently used, such as hypo-fractionated external radiotherapy and partial breast irradiation by postoperative brachytherapy or by intraoperative radiotherapy (IORT). IORT allows to deliver radiation therapy in a single fraction during surgery. Two large, randomized trials (ELIOT and TARGIT-A) conducted in women aged 45 years and older showed higher rates of ipsilateral breast cancer recurrence (statistically significant only in ELIOT) without any differences in long-term overall survival in the IORT groups compared with the WBRT groups [4,5]. IORT is now considered an alternative to WBRT and an effective therapeutic de-escalation option in menopausal women with EBC and low risk for local recurrence [6].

Other randomized controlled trials showed a significant advantage of WBRT after BCS on local control compared to endocrine therapy alone but no benefit in terms of overall survival in older women with estrogen-receptor-positive EBC [7,8]. Based on these findings, the U.S. national guidelines recommended omitting adjuvant radiotherapy after BCS for EBC women 70 years of age and older who are prescribed endocrine therapy [9]. However, whether radiotherapy is beneficial in the elderly is still a matter of debate, with results of recent studies using observational real-world data reporting higher mortality rates in those who did not receive radiotherapy after BCS [10,11].

Since the French Cancer Plan 2014–2019, therapeutic de-escalation is encouraged to maintain the quality of life of patients with cancer and to promote rehabilitation and recovery [12]. Therapeutic de-escalation includes any attempts to modify the treatment by either making it shorter, less toxic, and less resource-demanding. However, IORT was only very recently (October 2021) mentioned by the health authorities as an option for selected EBC in women aged 50 years and older, and omission of radiotherapy is not currently a validated option [13].

Among radiotherapy de-escalation options, IORT is only available in a limited number of French cancer centers [14]. In this particular context, we implemented a qualitative exploratory study, which is part of a broader research program called CARTE (NCT05058196), interviewing EBC physicians of French comprehensive cancer centers (1) to describe their views on IORT as a de-escalation option for older women with EBC and (2) to explore their feelings about patients’ information needs in discussing this. The aim was to use these results to better understand physicians’ practices in term of therapeutic de-escalation and to help developing a patient decision aid comparing different radiotherapy options for women with EBC.

## 2. Materials and Methods

This qualitative study (exploratory phase of the CARTE prospective study) investigated attitudes and opinions of French physicians involved in EBC treatment on IORT as a de-escalation alternative to WBRT in older women. Semi-structured interviews were conducted with open-ended questions on IORT, de-escalation, and patients’ information needed to participate in such decision making. Radiotherapy omission was also systematically discussed as proposed in several countries [9].

### 2.1. Data Collection

Recorded face-to-face and telephone semi-structured interviews were conducted among physicians working in one of the 18 French comprehensive cancer care centers grouped in the national federation UNICANCER. All these centers provided WBRT, but IORT was only available in few centers with two techniques: radiotherapy with low-energy X-rays using the Intrabeam^®^ system and radiotherapy with low-energy electrons using devices such as Mobetron^®^ or Novac-7^®^.

In order to obtain the full spectrum of opinions toward alternatives to WBRT and the diversity of current professional practices, a maximum variation sampling strategy regarding the availability or non-availability of IORT in the center and medical specialty was chosen. 

Mobilization of the members of UNICANCER’s national radiotherapy committee (UNITRAD) and the launch of a national call for volunteers sent by email to all eligible practitioners made it possible to implement the survey and facilitate access to interviewees. The same psychologist adopting the formal stance of deliberate “naïveté” to maintain open-minded attitudes and ensure spontaneous or unexpected answers conducted all interviews [15]. Race/ethnicity data were not collected, as it is not permitted in France except under special circumstances. The study was approved by UNICANCER CRP (RAD07–04/03/2019), and all participants provided oral consent.

### 2.2. Data Analyses

The analytic approach was based on semantic content analyses. Interview transcripts were grouped as corpus in order to design a typology of practitioner’s spectrum attitudes toward IORT (in favor opposed to against) and to describe their view of patients’ information needs. Given the specific aims of this study restricted to a description of opinions and practices about a medical cancer treatment, thematic analysis was performed using two stages of coding [16]. Following descriptive approach on physicians’ opinions, the first level of codes was generated using values coding the medical practices-related process. Data theming was used to label summarization of data [17]. Then, axial coding grouped codes under hierarchical and ordered umbrella “themes” to define and classify types of opinions and/or practices. Data saturation rules [18] and the triangulation method were used to ensure the quality of the coding process. To describe the variability of themes defined, a concept table and clustered matrix were used to display findings [19].

## 3. Results

Sixteen volunteer physicians, all caring for EBC patients, were interviewed between January and September 2019 (Table 1). They were breast surgeons (n = 4), radiation oncologists (n = 11), and medical oncologist (n = 1) from seven different comprehensive cancer centers throughout France. IORT technique was available in 5/7 centers, with three physicians working in a center without IORTC. Overall, none of the physicians interviewed considered radiation omission as a therapeutic de-escalation option (Appendix A).

### 3.1. Arguments in Favor or against IORT

Although the eligibility criteria for IORT were consensual (Appendix A), three groups of physician opinions emerged for this option: in favor (n = 5), not in favor (n = 4), and an intermediate position (n = 7). Emblematic quotes of advantages and disadvantages of IORT reported by the physicians are detailed in Appendix B. The occurrence of the different arguments used by the physicians is reported in Table 2. Physicians in favor of IORT considered it non-inferior and emphasized direct benefits for the patient in terms of decreased toxicity, constraints, and psychological impact. Those against it pointed out the risk of lost opportunity in older women with long life expectancy, the lack of specific guidelines, and the existence of other techniques with fewer side-effects than in the past. Physicians in a neutral position insisted on the importance of patient choice in a context where the positive effects for patients have to be balanced against a slightly higher local recurrence risk. They also highlighted the financial and logistical constraints for the hospital to explain that IORT could not be systematically proposed.

#### 3.1.1. Physicians in Favor of IORT (n = 5)

This group included three surgeons and two radiation oncologists. IORT efficacy, or at least non-inferiority compared to WBRT, was the most common favorable argument: “The studies on intraoperative radiotherapy have demonstrated non-inferiority…, it means that the difference in relapse risk between this technique and the traditional one, is considered statistically equivalent. So, it means that today we have not demonstrated that we were putting the patients at an additional risk of relapse” (P8).

However, strict adherence to patient selection criteria is required for an effective treatment. IORT, as treatment de-escalation, has been described as particularly interesting and beneficial for older women with small tumors: “The population of women aged 70 and over is a good fit for de-escalation of treatment. There’s a lot more benefit for this population when you consider the risks/benefits” (P13). Another argument in favor of IORT was the opportunity of performing the entire treatment in one day, reducing the number of home–hospital trips, which are known to be very difficult for elderly. IORT was associated with less radiotherapy and transportation-related fatigue. Another frequent argument was the excellent tolerance and low treatment-related toxicity. The precision of the irradiated area was also cited as a clinical advantage of IORT as well as the possibility to propose a conservative treatment in case of local relapse, which is less possible with WBRT (P8).

Although strongly in favor of IORT, physicians also pointed out some disadvantages such as increased time to initiate treatment and longer operating time. The fact that additional radiotherapy is required in 10–50% of cases is another disadvantage that must be well explained to patients, as its negative psychological impact may be important. Some physicians minimized this disadvantage, considering it as a form of patient-specific treatment adaptation: “It is a constraint for the patients, but it is also a security… If after the surgery we need to add radiotherapy, there is no loss of effectiveness, but only an increase in the duration of treatment” (P15); “With IORT, we can perfectly adjust postoperatively if there is a need to do more. So, whether it lasts one hour or 5 weeks, the patient will have a treatment that corresponds to her case. That’s how you can see the treatment, as adaptive” (P8). All physicians considered that IORT considerably reduces constraints for most patients but increases constraints on the medical team and the hospital because the cost of IORT is not reimbursed by national health insurance and therefore fully covered by the hospital.

#### 3.1.2. Physicians Not in Favor of IORT (n = 4)

All of the physicians were radiation oncologists. The most frequent argument against IORT was its potential reduced effectiveness in terms of long-term risk of recurrence compared with WBRT. One physician who used this argument relied on recent European guidelines emphasizing the higher risk of relapse with IORT in the ELIOT trial [20] (P4). The difficulty in selecting patients was also mentioned, as the elderly population is heterogeneous in terms of geriatric status and comorbidities: “The elderly population is very heterogeneous, women in their 70s are young, you can’t take risks, it all depends on the prognosis” (P5). Furthermore, the single session of radiotherapy may be misinterpreted by patients who may believe their cancer is less severe, leading to poor compliance with adjuvant endocrine therapy although such treatment must be taken after IORT: “A partial irradiation of the breast has to be associated with anti-hormonal treatment. The problem is that the patients who benefit from IORT have the impression that they have a small cancer, less aggressive than another, and so they say to themselves that finally the hormonal treatment, why take it, why bother me with it, with side effects?” (P11).

Physicians not in favor of IORT considered that WBRT techniques have considerably improved recently: “WBRT is responsible of far fewer side-effects than in the past, both in the short and long term, including a currently minimal risk of pulmonary and cardiac complications” (P5). One physician referred to a recent study showing that standard radiotherapy was well tolerated in older women and did not impair quality of life [21]. According to P3, local intolerance reactions are even less common in older than younger women, and daily trips to and from the hospital are only problematic for very old women (≥80 years). Moreover, hypo-fractionated radiotherapy (15 sessions in three weeks) is increasingly used in older women, better tolerated than the 5-week radiotherapy (P3, P5), and considered the standard procedure in the elderly (P5). Two physicians also reported the convenient use of brachytherapy as partial breast irradiation (P3, P4).

The last argument against IORT concerned the organizational constraints of this technique, as it requires simultaneous availability of the radiation therapist, physicist, surgeon, and anesthetist. Because of this, one radiation therapist decided to stop using IORT (P11).

#### 3.1.3. Physicians in a Neutral Position (n = 7)

Among the seven physicians in this group, there were five radiation oncologists, one surgeon, and one medical oncologist. They all worked in hospitals where IORT was available, and some of them used it.

The majority were not opposed to IORT but believed that this treatment option should be considered with caution due to slightly higher risk of local recurrence (P6), poorly known long-term risk (P10), and the absence of validation by the health authorities outside clinical research. However, most considered that IORT should be offered to eligible patients although physicians need time to explain the treatment options and their consequences to the patients who should then be given additional time to make their decision: “The problem with patient education is that it is very time consuming, so consultations usually last more than half an hour when we start talking about everything. It takes time and it takes time for the patients to digest the information. I think what I’m missing today is a pre-therapy consultation” (P12).

For all the physicians, one of the main advantages of IORT is that the entire treatment is done in one day. This technique also allows to take into account the social context, particularly for elderly who tend to restrict their treatments to take care of their husbands (P9).

IORT was considered well tolerated with less frequent and serious side effects compared to WBRT. However, some described more postoperative remodeling in months following surgery (P1) and more lymphoceles in the surgical bed (P2). The most cited disadvantages were the (1) waiting period (days) before knowing if additional radiotherapy is needed and (2) the systematic adjuvant endocrine therapy after IORT.

Finally, physicians mentioned hypo-fractionated radiotherapy and brachytherapy, which are widely used in >70-year-old women and minimize the risk of recurrence (P6). Compared with other techniques, the lack of reimbursement by health insurance would be a real limit to the use of IORT (P6).

### 3.2. Physicians’ Views on Information Need and Decision Making for IORT

Most physicians believed that the doctor’s opinion is the most important factor in the patient’s choice to choose or to refuse the treatment. One physician pointed out that patients do not have enough time to choose between several treatments: “Patients are given the information, it takes time. There should be a cooling off period. It would require a new consultation, meaning we couldn’t schedule treatment at that time... So yes, they are making a choice, but it’s an immediate choice. They can always say, “No, I don’t want that, I want the standard treatment,” but the question does not really come up. Maybe it’s because they’re not given time to think about it, maybe it’s because the information is not clear enough” (P16).

According to the physicians, women mainly accept IORT because of the reduction of both logistical constraints and expected side effects, allowing them to maintain their quality of life. The one-day treatment reduces travel time as well as stress and fatigue associated with waiting times. This is often positive for women but not for those who need to be reassured with maximal treatments: “There are other patient profiles who are more worried, and who are more demanding to have the maximum of things, they are more reassured to have a maximum treatment” (P5). Among the reasons why women might refuse IORT, physicians mainly mentioned adjuvant endocrine therapy, fear of relapse and the choice of the treatment starting as early as possible, refusal to participate in a clinical study, or fear of being alone in the operating room for the duration of the irradiation. Finally, one physician mentioned a poorer aesthetic result.

Moreover, WBRT suffers from a bad public image that may lead women to choose another treatment option if offered. In this case, there is a real impact of media in the women’s choice: “After a television report on IORT, there is a peak in requests”(P8); “Since IORT is not available everywhere, some patients come from far away to have this treatment”(P15). Conversely, a bad experience among relatives may cause patients to refrain from using specific therapeutic options such as IORT. For additional emblematic quotes, see Appendix C.

## 4. Discussion

In this study, only five of the sixteen French oncologists surveyed were strongly in favor of de-escalation by IORT, and none were comfortable with omitting radiotherapy after breast-conserving surgery for EBC. Our findings suggest that therapeutic de-escalation remains a sensitive topic in the context of EBC management in the elderly in France.

### 4.1. Limits and Strengths

Recruitment of volunteer physicians mainly working in hospitals where IORT was available was a limitation in this study. Some practitioners may have declined to participate due to the lack of consensus on partial irradiation techniques and the limited availability of IORT. However, the opinions collected give us a good understanding of the difficulties of implementing shared decision making in this context of de-escalation.

### 4.2. Treatment De-Escalation or Therapeutic Adaptation?

De-escalation aims is to reduce treatment toxicity while maintaining cancer control. De-escalation is therefore fundamentally linked to a patient-centered approach to maintain quality of life. In this study, all participating physicians were familiar with IORT and its indications even if not all of them had IORT available in their center.

As shown in Table 2, physicians who were in favor or less supportive of IORT used different arguments insisting on the perceived advantages or disadvantages for the patients but also taking into account the impact on the organization of care as compared with other techniques. Physicians in favor of IORT spontaneously defined it as therapeutic de-escalation. They all worked in a center equipped with IORT and systematically proposed it to eligible women. IORT was presented as a treatment option linked to good prognosis, and the emphasis was placed on the benefit for the patient in terms of fatigue, fewer side effects, or treatment duration. Conversely, physicians who were opposed to or less supportive of IORT did not mention therapeutic de-escalation. They presented WBRT as the standard treatment and other radiotherapy options as alternatives only for patients not eligible to WBRT for medical (frailty, co-morbidities, loss of autonomy) or social (isolation, travel difficulties) reasons. In this context of treatment adaptation, IORT was described as one therapeutic option to shorten the course of the treatment and radiation exposure in the same way as hypo-fractionated external radiotherapy or partial breast irradiation by postoperative brachytherapy. Moreover, they argued that hypo-fractionated external radiotherapy was increasingly considered the standard procedure for older patients.

### 4.3. Barriers to Treatment De-Escalation

Although IORT was considered a convenient technique for patients in terms of duration and impact on quality of life (one-day treatment), the reluctance expressed towards IORT was mainly related to reduced effectiveness to prevent risk of local relapse compared to WBRT or other partial breast irradiation techniques [3]. Most physicians did not want to opt for a treatment that puts their patients at greater risk of relapse. This is consistent with the results of an Australian survey showing that in a hypothetical scenario, most health professionals were willing to accept IORT (instead of WBRT) for themselves only if the maximum increase in risk of local recurrence did not exceed 3% [22].

The other limitations to consider IORT as an option were the lack of scientific consensus and national recommendations although clinical practice guidelines developed by health organizations and oncology societies [3] are available at the local level to ensure fair breast cancer care for all. A recent population-based study showed a good compliance of French physicians with clinical guidelines for breast cancer management [23]. However, there is a gap between general health policies promoting therapeutic de-escalation and personalization of cancer care [12] and French professional guidelines. According to the latter, radiation omission is not an option after BCS, and IORT has been very recently recommended (after this survey was performed) for highly selected patients [13]. In our study, there is no consensual agreement between physicians for IORT, and none of them were willing to propose radiation omission even in the context of clinical trial and knowing that it was considered a therapeutic option in several countries [9]. Of note, even in countries that recommend radiation omission as a de-escalation option, there is no consensus among radiation oncologists and surgeons. Many U.S. physicians tend to overestimate the risk of locoregional recurrence in older women with omission of radiotherapy and still recommend radiotherapy [24]. In the French medical culture, outcomes often take precedence over the patient’s wishes. However, recent results might help considering targeted radiation omission as a de-escalation option [25].

Another hurdle is that IORT can only be offered to women from the age of 55. This notion of age was problematic for several physicians. Geriatric disabilities are highly prevalent in older cancer patients, and geriatricians strongly recommend taking them into account when making therapeutic decisions, as they impact life expectancy irrespective of age [26]. In line with geriatric recommendations, reducing treatment was often seen as a positive adjustment in very old women or those with severe co-morbidities, but some physicians saw it as a potential loss of opportunity for “healthy” patients over 70. In a recent U.S. survey [27], women aged 70 years or older expressed identical skeptical views regarding age-based breast cancer treatment guidelines, considering that they should not apply to healthy older women with long life expectancy.

Moreover, material and organizational constraints are also to be taken into account, particularly the high cost of the equipment needed to offer IORT and the significant financial burden supported by hospitals since this treatment is not covered by the National Health Insurance.

### 4.4. Challenges for Shared Decision-Making

For the physicians, the higher risk of relapse with IORT could outweigh the benefits of “lighter” treatment and requires that the patient understand detailed information on the benefits/risks ratio. Most physicians stressed the need for shared decision making in this context but also the practical difficulties of involving patients in this choice. Patients should have accurate understanding of prognosis (increased risk of local relapse without impact on survival) and of potential additional radiotherapy (with associated “multi-modal” risk of complications) and mandatory adjuvant endocrine therapy when IORT is proposed. Many mentioned that the format of consultations does not allow physicians to give the patient the information required and the patient sufficient time to make informed decision. In line with a wide range of shared decision-making hurdles and facilitators across individual, organizational, and system levels reported in hospital setting [28,29], they also mentioned the influence of relatives and media. Moreover, uncertainties about the prognosis may hamper doctor–patient communication [30,31].

The reduction of expected side effects as well as the one-day treatment (instead of 3–5 weeks) were considered by physicians as important factors in women’s choice of treatment. These results are consistent with those of various studies conducted in France [32] and abroad [33], showing that older women with EBC tend to prioritize their independence and quality of life when they have to choose between several treatments. However, most physicians, including some in favor of IORT, believed that their own opinion was the major factor influencing the patient’s choice. This particularly suited physicians who also had a paternalistic view of the doctor–patient relationship. Treatment de-escalation chosen by the physician might meet the preferences of older women with EBC [34,35]. However, the latter are generally not satisfied with a paternalistic decision making and want to obtain further information about different treatment options before making their final choice [32,33,36].

## 5. Conclusions

Despite national policies promoting better preservation of the quality of life of cancer patients and their increased participation in medical decision making, de-escalation is not systematically discussed in French medical guidelines. Tailoring treatment, especially in elderly patients with cancer, has become common practice, but treatment de-escalation is less familiar and less consensual among physicians. Physicians should guide each patient through the best therapeutic options considering tumor characteristics, age, comorbidities, patient values, and wishes. Further efforts are needed to promote patient-centered care. More research would help to more accurately understand which patients can benefit from a personalized approach in search of the optimal benefit/risk ratio. The more systematic use of patient decision aids by trained physicians may help patients to consider different options and promote their meaningful participation in decision-making.

## Figures and Tables

**Table 1:** Characteristics and opinions of the interviewed physicians.

Physician	Gender	Medical or Surgical Specialty	Working in a Cancer Center with IORT	Opinion on IORT
P1	F	Radiation oncologist	Yes	Neutral
P2	F	Radiation oncologist	Yes	Neutral
P3	F	Radiation oncologist	No	Not in favor
P4	M	Radiation oncologist	No	Not in favor
P5	M	Radiation oncologist	No	Not in favor
P6	M	Radiation oncologist	Yes	Neutral
P7	F	Breast surgeon	Yes	In favor
P8	F	Radiation oncologist	Yes	In favor
P9	F	Medical oncologist	Yes	Neutral
P10	M	Radiation oncologist	Yes	Neutral
P11	F	Radiation oncologist	Yes	Not in favor
P12	M	Breast surgeon	Yes	Neutral
P13	F	Breast surgeon	Yes	In favor
P14	F	Radiation oncologist	Yes	Neutral
P15	F	Radiation oncologist	Yes	In favor
P16	M	Breast surgeon	Yes	In favor

IORT, intraoperative radiotherapy.

**Table 2:** Occurrence of the physicians’ arguments about IORT.

Arguments for or Against IORT		In Favor of IORT	Neither for Nor Against	Not in Favor of IORT
Requires strict patient selection		+++	++++	+
Treatment efficacy	Non-inferiority compared to conventional radiotherapy	+++		
	Difficulty to assess the risk of local recurrence	+	+++	+
	Higher risk of local recurrence		+++	++
Duration of the treatment	One-day treatment	++	+++	
	Extension of the operating time	+	+	+
	Treatment not always definitive	+	+	+
Side effects and sequelae	Well-tolerated treatment with less toxicity	+++	++++	
	But sequelae anyway		+	++
Psychological impact	Positive	++	+	
	Negative			+
Requires compliance with hormone therapy		+	+	+
Logistical constraints	Increased logistical constraints for hospital and physicians	+	+++	++
	Decreased logistical constraints for patients	+++	++++	
The financial cost is born by the hospital			++	
Other techniques of partial breast irradiation			++	+++

## Data Availability

The datasets generated during the current study are available from the corresponding author on reasonable request.

## Appendix A. Physicians’ View on Clinical Indications and Expected Side Effects of Intraoperative Radiotherapy (IORT) and Radiation Omission

IORT: A broad consensus was expressed by physicians about clinical indications of IORT: it may be an alternative to whole-breast adjuvant radiotherapy (WBRT) for postmenopausal women over 55 years of age, with nonspecific, ductal, grade 1 or 2 carcinomas, unifocal tumors of good prognosis and size <20 mm, no lymph node involvement, hormone receptor positive, and HER2 not overexpressed. If the cancer is “in situ”, it must not be expansive. Unifocal character of the tumor must be confirmed by angiomammography and MRI before treatment can be proposed. However, according to participants, other criteria may be taken into consideration to offer this treatment: geographical remoteness of the patient, very old age and lower life expectancy, other more serious cancer (allows treatment of everything at the same time) and previous radiotherapy (P15), technical contraindication or impossibility of WBRT (impossibility to lift the arms for example) (P1), and associated comorbidities or severe disease making external radiotherapy of little benefit compared to IORT (P11). Most physicians also considered that there are fewer side effects and sequelae with IORT than with WBRT: less fatigue, less breast sequelae (less fibrosis, burns, pain), better cosmetic result (less breast deformity), and less long-term toxicity to peripheral organs (lung and heart).
Radiation omission: In the case of breast-conserving surgery, withholding radiotherapy was not considered a treatment option by the physicians interviewed, mainly because it is not currently an option validated by the French health authorities, and it is associated with an increased risk or at least uncertainty about long-term risk of cancer relapse. As a result, radiation omission was exceptional and was only described in very old patients with poor general conditions, major co-morbidities, or short-term vital prognosis or when it was technically impossible to carry out radiotherapy. According to the interviewed physicians, radiation omission could only be an option in conjunction with radical mastectomy. Some doctors regretted that women were not well informed about this treatment option, as some might choose total mastectomy, which avoids radiotherapy while ensuring optimal local control. The physicians interviewed were rarely confronted with patients refusing radiotherapy. Refusal may be related to fear of radiation in women who have a breast prosthesis or in the oldest patients who do not invest much in the future and are afraid of all the treatments. It may also follow a history of very negative family radiotherapy experience or may be part of a broader context of overall refusal of conventional care in patients who turn to alternative medicine.

## Appendix B. Physicians’ Opinions about IORT

**Arguments**	**Emblematic Quotes**
Advantages of IORT	
Non-inferiority compared to WBRT if strict adherence to patient selection criteria	“To compare efficacy, we rely on published studies which show a very marginal benefit in favor of standard treatment. The selection of patients in these studies was perhaps a little less strict than what we do...We take a lot of precautions to have an equivalent benefit between the two treatments. With very precise criteria, the two treatments are equivalent.” (P15)
Precision of the irradiated area	“In WBRT there is radiotherapy of the whole breast and then there is over-irradiation localized on the area of the excision. With IORT, we are sure that the boost, is done exactly where it should be done.” (P16)
One-day treatment	“Avoiding round trips and transportation is a big advantage for patients who are more vulnerable. The benefit in terms of quality of life is clear.” (P1)
Well-tolerated treatment and low toxicity	“Toxicity is less at the skin level and at the level of peripheral organs.” (P13) “The breast remains supple, it is not fibrotic, we don’t have the problems of burning that we have with external radiotherapy.” (P7) “There is less deformation of the breast, especially in elderly women, who often have significant late deformation of the breast treated with conventional radiotherapy.” (P8)
Positive psychological impact for the patient	“For the patient, it’s psychologically more positive to have a one-day treatment, meaning that you arrive in the morning and go home after the surgery. There is just a continuous oral treatment to take, and the psychological impact is much less than with WBRT, and the patients have an excellent experience.” (P13)
Important benefit for older women with small tumors	“We have been slow to explore therapeutic de-escalation and giving an 80-year-old woman with a 6-mm tumor, six weeks of radiation is heresy. This is really the concept of therapeutic de-escalation. For small cancers, these are patients for whom the Americans and the British say in their standards that we can do without radiation, with relatively high relapse rates. Rather than not doing radiation, let’s do targeted radiotherapy.” (P8)
Disadvantages of IORT	
Unknown long-term risk of recurrence and difficulty in selecting patients	“In highly selected patients, we do not feel that there is a greater risk of local relapse. Afterwards, we need a 10-year follow-up.” (P1) “This is a technique that is still being evaluated and we do not have that much experience with it.” (P12)
Need of extra time to share decision with the patient	“Initially I was trying to present things in a very neutral way by really explaining even the uncertainties of medicine, because we have plenty of them, and in fact patients don’t expect that at all. They want to be guided… even if we make them understand that it is a choice, this choice must be guided.” (P14)
Delay to initiate treatment	“Conventional treatment which consists of an outpatient lumpectomy and sentinel node sampling, can usually be completed within 15 days. If IORT is chosen, an angio-mammogram, an MRI and a consultation with the radiation therapist are required, which takes more time. The date of the breast surgery is necessarily delayed compared to the one that could be proposed. This is not serious from an oncological point of view for tumors with a good prognosis, but it is sometimes a little complicated to manage with patients.” (P13)
Additional radiotherapy often needed	“It takes about ten days to get the results and to know whether or not additional radiotherapy is needed... I always approach the consultation with the patients by saying: we propose the treatment but, in any case, what counts is the definitive analysis and as long as we don’t have that, we can’t affirm that you won’t need another postoperative treatment.” (P15)
Negative psychological impact for the patient	“That’s the psychological problem of the IORT. We tell the women it’s nothing at all, the little lady sees the surgeon, we operate on her, we don’t really talk about radiation, we wake her up, and then finally we announce that we have to do radiation... it’s certain that in this case, she’ll say to herself that it’s perhaps much more serious, and there’s anguish.” (P8) “If the IORT is not validated and additional radiation therapy is required, women are extremely disappointed. The disappointment and the psychological impact are greater than for a patient scheduled for conventional radiotherapy and who must have more sessions.” (P7)
Compliance with adjuvant endocrine therapy needed	“They absolutely have to comply with the anti-hormonal treatment, and we don’t have the key to know if they will take it knowing there are problems of compliance to hormone therapy for breast cancer. We need to make women accountable for this.” (P14)
Specific side effects	“The inflammatory reactions are stronger with intraoperative radiotherapy. As an immediate reaction, there may be greater discomfort, redness, pain and inflammatory effects. When patients are a little obese, they have more skin reactions and pain” (P9)
Increased logistical constraints for hospital and physicians	“Even when the organization is satisfactory, IORT extends the operating time. This can be a constraint for the anesthesia team, for the surgeons. Normally it lasts about three quarters of an hour to an hour longer, so it is a time to be considered for the occupation of the room and the occupation of the staff.” (P15)
No benefit compared to other techniques	“With hypo-fractionated radiotherapy there is less fatigue, less travel, no increased delay in care. It is widely used in some centers for elderly women. Duration 3 weeks and one day.” (P3) “For patients over 60 years, with good prognosis tumors, we propose local brachytherapy, i.e., a shorter treatment, which is less irradiating, which targets the operating bed.” (P4)
	WBRT, whole breast radiotherapy; IORT, intraoperative radiotherapy.

## Appendix C. Physicians’ Views on Factors in Women’s Decision Making for IORT

**Factors**	**Emblematic Quotes**
Supporting or opposing factors	
Opinion of the physician	“When the doctor is convinced of the benefit of the treatment, his or her opinion strongly influences the patient’s choice.” (P5) “Some colleagues may be more reticent and so I think that this plays into the patient’s choice.” (P15) “Some patients don’t want to choose and when the different solutions are presented, they are lost and say: no, I trust you and will do what is best for me.” (P12)
One-day treatment	“When fewer sessions are offered, patients are immediately willing to accept.” (P1)
Public image and experience among relatives	“Conventional radiotherapy is a treatment that is still frightening, that is a bit mysterious, patients don’t know what to expect. There is a lot of talk about the side effects, so it is a treatment that is sometimes misunderstood, with a negative attitude on the part of patients. Possibility of not doing postoperative conventional radiotherapy can influence the choice.” (P15) “Women are influenced by what they have seen, heard, about the different techniques, what some people around them may have told them as a personal experience, a bad experience of external radiotherapy or IORT.” (P11)
Supporting factors	
Preservation of quality of life	“Older women will opt for the least burdensome, least aggressive treatment that has the least impact on their autonomy. Older patients want to remain independent”. (P9) “One of the criteria that can influence patients is their own experience and what they expect from the treatment in terms of its impact on their quality of life.” (P11)
Less travel time	“It is difficult to have a medical transport…the transporter is not under control, that is stressful for the patient… in the context of radiotherapy there may be unexpected waiting times, you are scheduled for a given time, but this is shifted for reasons of patient flow, independently of everyone’s goodwill. You wait for an hour, a few minutes under the machine, you wait again for your ambulance driver, and then you are back home. In fact, the days are centered on the half-day needed to do all this.” (P9)
Opposing factors	
Fear of relapse	“I have patients who tell me, I want the whole thing, the longest, heaviest treatment, because I am afraid.” (P15)
Delay before treatment initiation	“With IORT, the delay in management is sometimes longer, which can lead to a refusal of the intraoperative procedure and the choice of the treatment that begins most quickly.” (P13)
Refusal of adjuvant endocrine therapy	“There are many women who for X or Y reasons do not want anti-hormonal treatment or do not take it correctly, I insist on the fact that it is the whole procedure that allows a better chance of cure.” (P15) “I propose IORT with the only constraint that the woman agrees to take the anti-hormone therapy. This seems to be the major determinant, and this is what I insist on a lot with patients, saying that I agree to do the IORT, but if they agree to take this anti-hormonal treatment. If they are reluctant at the beginning, I think it is not a good treatment because the few cases of relapse that we have had were in patients who had not taken the anti-hormonal treatment.” (P11)
Poorer aesthetic result	“To install the intrabeam, the surgeon must make a direct approach. If you have a tumor on the edge of the breast, you cannot go through the nipple, you will have to go directly, which is not as pretty… in women aged 55-60, I have seen some recently who have refused the intraoperative procedure for this reason.” (P13)
